# Polyhydramnios in *Lrp4* knockout mice with bilateral kidney agenesis: Defects in the pathways of amniotic fluid clearance

**DOI:** 10.1038/srep20241

**Published:** 2016-02-05

**Authors:** Hiroshi Tanahashi, Qing-Bao Tian, Yoshinobu Hara, Hiroyuki Sakagami, Shogo Endo, Tatsuo Suzuki

**Affiliations:** 1 Department of Neuroplasticity, Institute of Pathogenesis and Disease Prevention, Graduate School of Medicine, Shinshu University, 3-1-1 Asahi, Matsumoto 390-8621, Japan; 2 Department of Biological Sciences for Intractable Neurological Diseases, Institute for Biomedical Sciences, Shinshu University, 3-1-1 Asahi, Matsumoto 390-8621, Japan; 3 Department of Anatomy, Kitasato University School of Medicine, 1-15-1, Kitasato, Sagamihara, Kanagawa 252-0374, Japan; 4 Research Team for Aging Neuroscience, Tokyo Metropolitan Geriatric Hospital and Institute of Gerontology, Sakae-cho, Itabashi, Tokyo 173-0015, Japan

## Abstract

Amniotic fluid volume during mid-to-late gestation depends mainly on the urine excretion from the foetal kidneys and partly on the fluid secretion from the foetal lungs during foetal breathing-like movements. Urine is necessary for foetal breathing-like movements, which is critical for foetal lung development. Bilateral renal agenesis and/or obstruction of the urinary tract lead to oligohydramnios, which causes infant death within a short period after birth due to pulmonary hypoplasia. *Lrp4*, which functions as an agrin receptor, is essential for the formation of neuromuscular junctions. Herein, we report novel phenotypes of *Lrp4* knockout (*Lrp4*^−/−^) mice. Most *Lrp4*^−/−^ foetuses showed unilateral or bilateral kidney agenesis, and *Lrp4* knockout resulted in polyhydramnios. The loss of *Lrp4* compromised foetal swallowing and breathing-like movements and downregulated the expression of aquaporin-9 in the foetal membrane and aquaporin-1 in the placenta, which possibly affected the amniotic fluid clearance. These results suggest that amniotic fluid removal was compromised in *Lrp4*^−/−^ foetuses, resulting in polyhydramnios despite the impairment of urine production. Our findings indicate that amniotic fluid removal plays an essential role in regulating the amniotic fluid volume.

Amniotic fluid provides an optimal environment for normal foetal growth and development. During the early stages of pregnancy, amniotic fluid is secreted from amniotic epithelium or chorion leave and absorbed through the chorioamniotic membrane. During mid-to-late gestation, the amniotic fluid is mainly composed of foetal urine and partly of foetal lung fluid, which is intermittently discharged from the lung alveolar spaces into the amniotic cavity during foetal breathing-like movements. Five potential mechanisms underlying the movement of amniotic fluid into the amniotic cavity have been described in chronically catheterized late-gestation ovine foetuses. Among these three pathways involved foetal urine (1010 ml/day), foetal lung liquid (380 ml/day), and fluid secreted from the foetal oral/nasal cavities (28 ml/day)^1^. The remaining two potential pathways involved negligible flow across the foetal skin and umbilical cord surface, therefore these were no longer relevant during late gestation, after skin keratinization^2^. The three relevant pathways of amniotic fluid removal were further investigated in late-gestation ovine foetuses. The main routes of amniotic fluid removal were foetal swallowing (580 ml/day) and intramembranous absorption (810 ml/day), which involves the transfer of amniotic fluid across the amnion and into the underlying foetal vasculature^1^. Transmembranous absorption, which refers to the transfer of amniotic fluid across the amnion/chorion into the maternal vasculature, appeared to be negligible during late gestation^1^,^2^. Disruption of any of these processes possibly results in abnormally low or high amniotic fluid volume, referred to as oligohydramnios or polyhydramnios, respectively. Oligohydramnios and polyhydramnios have been associated with increased perinatal morbidity and mortality^1^,^2^,^3^,^4^. In humans, amniotic fluid volume depends mainly on the foetal urine production after 16 weeks of gestation. Urine is necessary for foetal breathing-like movements, which is critical for foetal lung development, and it aids in supplying proline, a critical amino acid for lung maturation. Bilateral renal agenesis and/or obstruction of the urinary tract cause oligohydramnios, resulting in the death of infants within a short period after birth due to pulmonary hypoplasia^4^.

*Lrp4* is a member of the low-density lipoprotein (LDL) receptor gene family. We previously reported that the expression of the *Lrp4* protein was higher in the forebrain of adult rats than that in the other tissues examined^5^, although *Lrp4* expression has been detected in a number of organs at various developmental stages^6^,^7^,^8^,^9^. In muscle, *Lrp4* serves as a receptor for agrin and is essential for the activation of muscle-specific tyrosine kinase (MuSK) and the clustering of acetylcholine receptors, thus leading to the formation of neuromuscular junctions^8^,^10^,^11^. Mice bearing functional *Lrp4*-null mutations die at birth because it fails to form neuromuscular junctions^8^. However, mice with hypomorphic receptors, generated by inserting a stop codon upstream of the transmembrane domain of the *Lrp4* gene, are viable^7^. The loss of *Lrp4* in mice has been shown to result in fully penetrant polysyndactyly in the fore and hind limbs^7^,^8^, partially penetrant abnormalities in teeth^7^,^8^,^12^, impaired bone growth and increased bone turnover^9^, delayed ureteric budding and partially penetrant kidney agenesis^8^,^13^, and decrease in the lung size^8^. A similar polysyndactyly phenotype has been reported in cattle with other naturally occurring allelic mutations^14^,^15^,^16^. In humans, *LRP4* mutations have been implicated in Cenani-Lenz syndrome, an autosomal recessive congenital disorder causing facial dysmorphism, syndactyly, synostosis and kidney anomalies^17^, as well as congenital myasthenia^18^. Furthermore, autoantibodies against *LRP4* have been associated with myasthenia gravis^19^.

In the present study, we report the novel phenotypes of *Lrp4* knockout mice (*Lrp4*^−/−^) generated in this study. As expected, *Lrp4*^−/−^ mice died at birth due to their inability to breathe, and 69% of *Lrp4*^−/−^ mice displayed bilateral or unilateral kidney agenesis. *Lrp4*^−/−^ foetuses showed polyhydramnios in the perinatal period. The coexistence of polyhydramnios and bilateral kidney agenesis represented an unexpected finding, since foetal urine, a major source of amniotic fluid during the perinatal stage, is not produced by the kidneys. Therefore, we investigated the mechanisms underlying polyhydramnios observed in *Lrp4*^−/−^ mice with bilateral kidney agenesis.

## Results

### Generation of *Lrp4* ^−/−^ mice

**Figure 1:**
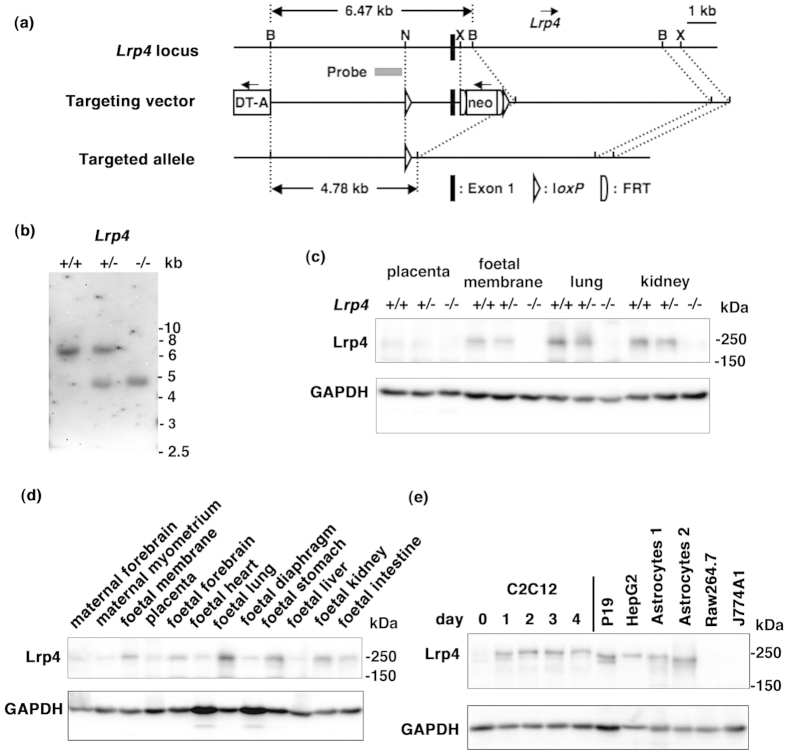
Targeted disruption of the mouse *Lrp4* gene. (**a**) Schematic illustration of the *Lrp4* gene, targeting vector, and targeted allele. The gray bar indicates the location of a probe for southern blot analysis. Abbreviations: DT-A, diphtheria toxin-A gene; neo, neomycin phosphotransferase gene; B, *Bam*HI; N, *Nco*I; X, *Xho*I. (**b**) Southern blot analysis of genomic DNA from wild-type (*Lrp4*^+/+^), *Lrp4*^+/−^, and *Lrp4*^−/−^ mice. *Bam*HI-digested DNA hybridized with a probe. (**c**) Cell lysate proteins (40 μg protein/lane) from the placenta, foetal membrane, kidneys, and lungs of E18.5 *Lrp4*^+/+^, *Lrp4*^+/−^, and *Lrp4*^−/−^ mice were analyzed by western blot analysis with anti-LRP4 and anti-glyceraldehyde-3-phosphate dehydrogenase (GAPDH) antibodies. (**d**) Western blot analysis of *LRP4* expression in various tissue homogenates from E18.5 foetuses and a maternal mouse. (**e**) *LRP4* expression in various cells. C2C12, mouse muscle myoblast; HEPG2, human hepatocellular carcinoma; RAW264.7 and J774A.1, mouse macrophage; P19, mouse embryonal carcinoma. C2C12 cells were differentiated to myotubes by switching to differentiation medium for the indicated days. Two independent primary cultures (1 and 2) of astrocytes were obtained from mouse cerebrum at E18.5. The culture conditions of these cells are described in Supplementary information online. Since two western blot analyses showed similar results, one result was shown. Cropped blots are shown (full-length blots are presented in Supplementary Fig. S6 online).

**Figure 2:**
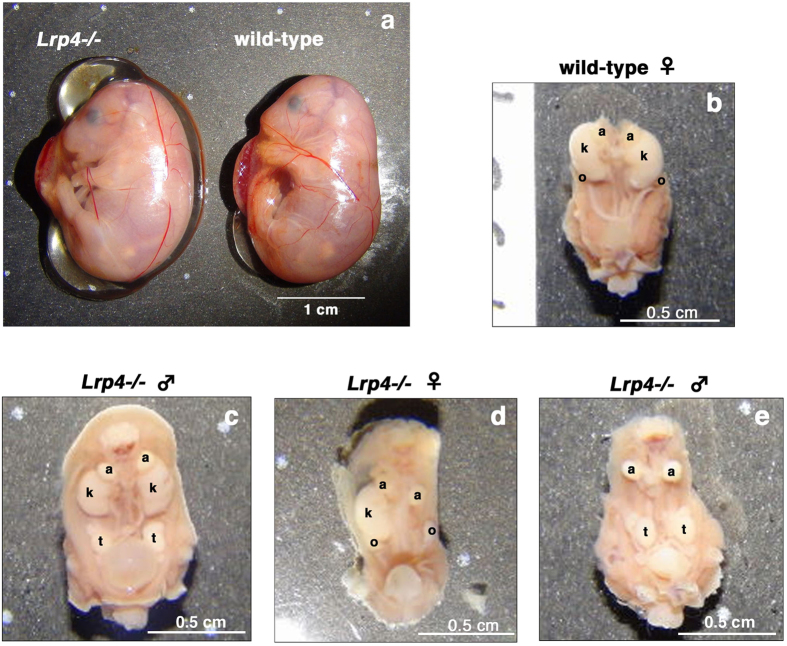
Polyhydramnios occurred in *Lrp4*^−/−^ embryos at E18.5. (**a**) Myometrium was removed from embryo. A representative *Lrp4*^−/−^ embryo with massive polyhydramnios and a littermate wild-type embryo are shown. (**c**–**e**) *Lrp4*^−/−^ foetuses (**c,d,e**) with bilateral (**e**) or unilateral (**d**) without (**c**) kidney agenesis and a wild-type littermate (**b**). a: adrenal gland, k: kidney, o- ovary, t: testis

A targeting vector was designed to replace exon 1 of the *Lrp4* gene with the *PGK* promoter-driven neomycin-resistance cassette (neo), by homologous recombination in murine embryonic stem cells (Fig. 1a). Heterozygous (*Lrp4*^+/−^) mice carrying an allele lacking *Lrp4* exon 1 and neo were generated as described in the Materials and Methods section. *Lrp4*^+/−^ mice were viable and fertile with no detected abnormalities, and their life spans were normal. Homozygous (*Lrp4*^−/−^) mice, obtained by crossing the heterozygotes, were born with a quasi-Mendelian trait; therefore, embryonic lethality was absent in these mice. *Lrp4*^−/−^ neonates exhibited polysyndactyly (Supplementary Fig. S1b online) and severe cyanosis, and died shortly after birth (Supplementary Fig. S1a online). Southern blot analysis (Fig. 1b), as well as sequencing analysis (Supplementary Fig. S2 online) confirmed successful targeting of the *Lrp4* gene (Fig. 1b). Immunoblotting revealed that *LRP4* was absent in the lungs and kidneys; it was rarely detected in the placenta and foetal membrane (the extraplacental chorioamniotic membrane) of *Lrp4*^−/−^ mice at embryonic day (E) 18.5 (Fig. 1c). We previously reported that the expression of *LRP4* was highest in the forebrains of adult rats, but was detectable also in the lungs, spleen, thymus, and testis^5^. However, in E18.5 mice, we observed that *LRP4* was expressed most highly in the lungs, with moderate expression in the kidneys, foetal membrane, stomach, forebrain, and intestine, and showed low expression in the diaphragm, heart, placenta, and liver (Fig. 1d). *LRP4* was expressed in various cells, including cultured astrocytes (Fig. 1e). Among *Lrp4*^−/−^ mice observed at E18.5 and E17.5, we found that 26% (77 of 295 *Lrp4*^−/−^ foetuses) exhibited bilateral kidney agenesis, whereas 43% (126 of 295 *Lrp4*^−/−^ foetuses) showed unilateral kidney agenesis (Fig. 2b). Unilateral renal agenesis was more frequently identified on the left side (58%, 53 right and 73 left; OR: 1.4, 95% CI: 0.8–2.3); however, this was not significantly different from the expected proportion (Fisher’s exact test *P* = 0.26). Similar findings have been reported for human unilateral kidney agenesis^20^. Visual inspection suggested that newborn *Lrp4*^−/−^ mice died due to respiratory insufficiency. The lungs of newborn *Lrp4*^−/−^ mice had four right lung lobes and a single left lobe flanking the heart, and were devoid of obvious abnormalities except for wet lung size (Supplementary Fig. S1c online). Lungs of wild-type and *Lrp4*^−/−^ mice were removed and placed in PBS^(−)^ (Supplementary Fig. S1d online). The lungs of wild-type mice were inflated and able to float, whereas those of the *Lrp4*^−/−^ mice did not float, suggesting that the latter did not inflate after birth. Microscopic analysis confirmed this conclusion (Supplementary Fig. S1e online).

### *Lrp4* ^−/−^ mice developed polyhydramnios during the perinatal period

Close-to-term *Lrp4*^−/−^ embryos (E17.5 and E18.5) showed polyhydramnios, and the weight of amniotic fluid in these mice was greater than in their wild-type and *Lrp4*^+/−^ littermates (Fig. 2a and Table 1). On the other hand, no significant differences were observed in the weights of foetuses and placentas among the three groups (Table 1). The osmolality of amniotic fluid was lower in *Lrp4*^−/−^ foetuses at E18.5 than in their wild-type and *Lrp4*^+/−^ littermates (Table 2). The major volume of amniotic fluid is generally considered to consist of foetal urine. We investigated whether bilateral and unilateral kidney agenesis affected the accumulation of amniotic fluid. Polyhydramnios was observed in *Lrp4*^−/−^ foetuses with bilateral kidney agenesis, and no significant differences were noted in amniotic fluid weights in *Lrp4*^−/−^ foetuses, regardless of the presence or absence of kidney agenesis (Table 1). Gastrointestinal atresia and stenosis, which affect amniotic fluid removal^4^, were not detected in *Lrp4*^−/−^ foetuses (Supplementary Fig. S3 online).

Twenty-seven percent (92 out of 295 *Lrp4*^−/−^ foetuses) of kidneys and urinary tracts formed in *Lrp4*^−/−^ foetuses were indistinguishable from those of the wild-type foetuses at the morphological level. Creatinine, produced naturally by the body, is freely filtered by the glomeruli of kidneys. Urea, a by-product of protein metabolism, is synthesized in the liver and removed from the blood by the kidneys. Therefore, urea and creatinine levels in urine serve as a primary measure of kidney function. Foetal waste products are primarily excreted by maternal kidneys through placental circulation^21^, and foetal kidneys start secreting urine during late gestation. Hence, analysis of creatinine and urea in amniotic fluid permits an evaluation of foetal renal maturation and functionality during late gestation^22^. We investigated kidney function in *Lrp4*^−/−^ foetuses at E18.5, and found that creatinine and urea concentrations in the amniotic fluid of *Lrp4*^−/−^ foetuses with bilateral and unilateral kidney agenesis were significantly lower than in their wild-type and *Lrp4*^+/−^ littermates. Additionally, we found that the concentrations of creatinine and urea in *Lrp4*^−/−^ foetuses without kidney agenesis were also slightly lower than those in their wild-type and *Lrp4*^+/−^ littermates (Supplementary Table S1 online). Renal maturation and functionality might have been impaired in the kidneys of some *Lrp4*^−/−^ mice that appeared to be indistinguishable from kidneys of wild-type mice at the morphological level, although histological studies would be need to confirm this. The change in all measured solute concentrations combined (sodium, potassium, chloride, urea, and creatinine) was small compared to the decrease in osmolality. This suggests that impairment of the entry of liquid from the lung into the amniotic fluid may play a role in the decrease in osmolality observed in *Lrp4*^−/−^ mice.

### Swallowing and foetal breathing-like movements were compromised in *Lrp4* ^−/−^ mice

**Figure 3:**
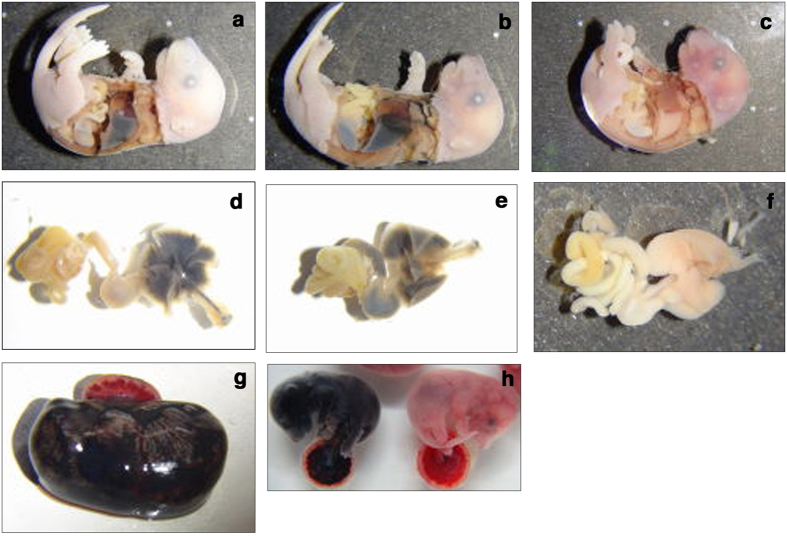
Foetal swallowing and breathing-like movements were compromised in *Lrp4*^−/−^ foetuses. India ink was injected into the amniotic cavity of embryos at E18.5. After 30 min, the amniotic membrane was ruptured, and the foetus and placenta were washed and fixed. Gastrointestine and respiratory tract (**d**–**f**) were took out from foetuses (**a–c**), respectively. Representative data are shown. India ink accumulation in the lungs of a wild-type foetus (**a,d**), and in the lungs and stomach of a wild-type foetus (**b,e**). India ink accumulation in the lungs and stomach was not observed in a *Lrp4*^−/−^ foetus (**c,f**). (**g**) Thirty min after injection of India ink into the amniotic cavity of a wild-type embryo, maternal myometrium was removed. India ink accumulation was not observed in the placenta of a wild-type embryo. (**h**) India ink was directly injected into the body of a wild-type foetus through the uterine wall (left) or injected into the amniotic cavity of a wild-type embryo (right). After 30 min, the amniotic membrane was ruptured, and the foetus and placenta were washed. India ink accumulation in the placenta was observed in the former, but not in the latter.

The two primary routes of amniotic fluid removal are foetal swallowing and intramembranous absorption. In order to evaluate swallowing, India ink was injected into the amniotic cavity of embryos at E17.5 and E18.5. After 30 min, the amniotic membrane was ruptured, and the foetus and placenta were washed and fixed. Accumulation of India ink was observed in the lungs and/or stomachs of wild-type and *Lrp4*^+/−^ foetuses, but not in *Lrp4*^−/−^ littermates (Fig. 3a–f and Table 3). However, accumulation of Indian ink was not detected in the placentas (Fig. 3g,h) of either the wild-type or *Lrp4*^+/−^ foetuses. These results suggest that carbon black colloids were not absorbed in the placenta, and that accumulation of India ink in the stomach and lungs did not occur via the placenta or foetal membrane. Accumulation in the lungs was attributed to foetal breathing-like movements that were detected from E14.5^23^. Foetal swallowing and breathing-like movements occurred more frequently at E18.5 than at E17.5 in wild-type and *Lrp4*^+/−^ foetuses (Table 3). These results demonstrate that foetal swallowing and breathing-like movements were compromised in *Lrp4*^−/−^ foetuses due to the failure to form neuromuscular junctions.

Since mechanical factors such as foetal breathing-like movements are known to be required for normal lung growth^24^,^25^, we measured lung weight and lung DNA content of *Lrp4*^−/−^ foetuses and their wild-type and *Lrp4*^+/−^ littermates. The wet lung weight to body weight ratio of the lungs of *Lrp4*^−/−^ mice at E18.5 was markedly lower (by 21%) than that of the lungs of wild-type and *Lrp4*^+/−^ mice, whereas no significant differences were observed in the dry lung weight to body weight ratio and lung DNA content among the three groups (Supplementary Table S2 online). Weatherbee *et al.* previously reported that *Lrp4* knockout mice exhibited a decrease in lung size (50–75%) and sacculation; however, no abnormalities in cell proliferation, cell death, or the expression of molecular markers of lung differentiation were observed, and organogenesis in the lungs of *Lrp4* knockout mice was found to be preserved^8^. These findings, as well as our results (Supplementary Table S2 online), suggest that the reduction in size of the wet lungs in *Lrp4*^−/−^ mice was due to reduced lung alveolar spaces and not due to reduced lung parenchyma.

**Figure 4:**
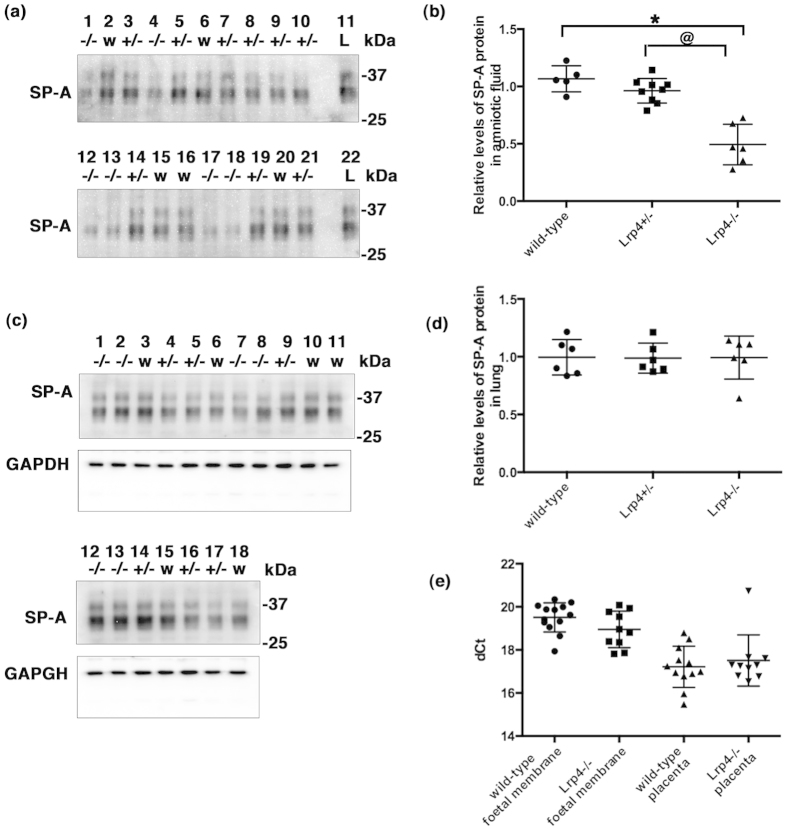
An *Lrp4* deficiency affected the secretion of the SP-A protein from the lung into the amniotic cavity. (**a**) Ten microliters of amniotic fluid (lanes 1–10, lanes 12–21) from wild-type (lanes 2, 6, 15, 16, 20), *Lrp4*^+/−^ (lanes 3, 5, 7, 8, 9, 10, 14, 19, 21), and *Lrp4*^−/−^ (lanes 1, 4, 12, 13, 17, 18) embryos at E18.5, and adult lung lysate (2 μg of protein) as a positive control (lanes 11 and 22) were analyzed by western blot analysis with anti-SP-A antibodies. Lanes 1–3, lanes 4–10, lanes 12–16, and lanes 17–21 were their respective littermates. (**b**) Amniotic fluid from *Lrp4*^−/−^ embryos contained significantly less of the SP-A protein than that from wild-type and *Lrp4*^+/−^ embryos. The mean of wild-type and *Lrp4*^+/−^ foetuses born of the same mother was set as 1, and each value of littermates was a relative ratio to the mean of wild-type and *Lrp4*^+/−^ littermates. *P<0.0001 versus the wild-type, @*P*<0.0001 versus *Lrp4*^+/−^ by Tukey-Kramer’s *post hoc* test after a one-way analysis of variance. Horizontal lines show the means ± SD. (**c**) Lung lysates (each 2 μg of protein) from wild-type (lanes 3, 6, 10, 11, 15, 18), *Lrp4*^+/−^ (lanes 4, 5, 9, 14, 16, 17), and *Lrp4*^−/−^ (lanes 1, 2, 7, 8, 12, 13) foetuses at E18.5 were analyzed by western blot analysis. After stripping, the same blots were analyzed using anti-GAPDH. Lanes 1,3,4, lanes 2, 5, 6, lanes 7–11, and lanes 12–18 were their respective littermates. (**d**) A quantitative analysis of (**c**) showed that the SP-A protein in foetal lungs did not significantly differ among the three genotypes. The SP-A protein expression levels were normalized to the GAPDH protein levels in the same sample. (**e**) A quantitative RT-PCR analysis for the expression of SP-A. An *Lrp4*- deficiency did not significantly change SP-A mRNA expression levels at E18.5 in the foetal membranes (wild-type n = 13, *Lrp4*^−/−^ n = 10) or placentas (wild-type n = 12, *Lrp4*^−/−^ n = 10). Cropped blots are shown (full-length blots are presented in Supplementary Fig. S6 online). w: wild-type, +/-: *Lrp4*^+/−^, ^−/−^: *Lrp4*^−/−^, L: adult lung lysate

Surfactant protein A (SP-A) is actively synthesized in the alveolar type II cells in the lungs, secreted into alveolar spaces^26^, and intermittently discharged into foetal amniotic fluid via foetal breathing-like movements^27^,^28^. In the foetal mouse lung, SP-A mRNA is detectable at day 15 of gestation^29^, and SP-A protein is detectable at E17 in the amniotic fluid^28^; these levels are found to increase progressively to term^28^. Therefore, we investigated the presence of the SP-A protein in the lungs and amniotic fluid of *Lrp4*^−/−^ mice by western blot analysis. The amniotic fluid of *Lrp4*^−/−^ mice showed significantly lower levels of SP-A protein than that in the wild-type and *Lrp4*^+/−^ littermates (Fig. 4a,b), whereas no significant differences were observed between the SP-A protein levels in the lungs (Fig. 4c,d) or the SP-A mRNA levels in the placentas and foetal membranes (Fig. 4e) of the *Lrp4*^−/−^, wild-type, and *Lrp4*^+/−^ mice. Since the SP-A protein is also expressed in the placenta and foetal membrane^28^,^30^,^31^, some of the protein detected in the amniotic fluid of *Lrp4*^−/−^ mice may have originated from the placenta and foetal membrane or a leak from the pulmonary alveoli. Therefore, we confirmed that foetal breathing-like movements were reduced in *Lrp4*^−/−^ mice.

### Expression of AQP9 and AQP1 was decreased in the foetal membrane and placenta

Previous studies proposed that four members of the aquaporin (AQP) family (AQP1, AQP3, AQP8, and AQP9) are involved in the control of amniotic fluid homeostasis^32^,^33^,^34^,^35^,^36^,^37^,^38^. In order to investigate the relationship between the expression of AQPs and polyhydramnios in *Lrp4*^−/−^ mice, we used RT-PCR to examine the expression of *Lrp4* in the mouse placentas and foetal membranes of mice at E13.5, E17.5, and E18.5. Screening for AQPs in the placentas and foetal membranes at E18.5 revealed the presence of AQP1, AQP3, AQP7, AQP8, and AQP9 mRNA (Supplementary Fig. S4 online). Quantitative RT-PCR for these AQPs showed that the expression of AQP9 mRNA was lower in the foetal membrane of *Lrp4*^−/−^ fetuses than wild-type foetuses on these three embryonic days, and the expression of AQP1 in the placentas of *Lrp4*^−/−^ foetuses at E17.5 and E18.5 was lower than that in the placenta of their wild-type littermates (Table 4, Supplemental Table S4 and Fig. S5 online). Therefore, *Lrp4* is considered to regulate the expression of AQP9 and AQP1, which may contribute to amniotic fluid volume regulation in mice.

## Discussion

Studies on the regulation of amniotic fluid volume have been performed mostly in humans and sheep, and foetal urine has been reported to be the main source of amniotic fluid during mid-to-late gestation^1^,^2^. Glomerular filtration and tubular maturation have been reported to begin at E14.5 in the kidneys of mice^39^. It has been suggested that amniotic fluid dynamics differ partially in humans and mice, since the predelivery decrease in amniotic fluid volume is greater in mice than in human^40^,^41^. Mice have not been extensively used for studies on amniotic fluid dynamics. To date, the source of murine amniotic fluid (e.g., urine, lung liquid, or plasma) at various gestational ages has not been determined.

In the present report, we describe novel phenotypes of *Lrp4*^−/−^ mice and present two possible causes of polyhydramnios observed in *Lrp4*^−/−^ mice: inability to swallow, which results in the disruption of a major route of amniotic fluid removal, and downregulated expression of AQP1 and AQP9, which may affect amniotic fluid clearance. Bilateral renal aplasia associated with oligohydramnios has been reported in Potter’s syndrome, which is also associated with lung hypoplasia^42^. Bilateral renal agenesis without oligohydramnios has been reported in newborn human infants^43^,^44^,^45^,^46^. In both cases, foetal swallowing and the reabsorption of amniotic fluid from the intestinal tract were disrupted due to anencephaly^43^ and oesophageal atresia^44^, respectively. Oligohydramnios did not occur in either case^45^,^46^, despite the congenital absence of both kidneys. The reason for this, and the non-renal origin of the amniotic fluid, remains obscure. Thus, amniotic fluid volume in foetuses appears not to be solely determined by the presence or absence of kidneys, but by multiple factors regulating the balance between supply and excretion of amniotic fluid. Myasthenia gravis is an autoimmune disease of neuromuscular junctions, in which autoantibodies against acetylcholine receptors, MuSK and *LRP4* have been observed in approximately 85%, 5%, and 2% of patients with myasthenia gravis, respectively^47^. Polyhydramnios has also been reported to occur in some cases of myasthenia gravis due to the impairment of foetal swallowing^48^,^49^,^50^, which was also observed in our *Lrp4*^−/−^ mice.

Our findings suggest that *Lrp4* regulates the expression of AQP9 and AQP1 (Table 4), which may contribute to the regulation of amniotic fluid volume in the perinatal period. Polyhydramnios has been found to develop in *Aqp1* knockout mice, along with a reduction in amniotic fluid osmolality^32^,^33^. This finding was not contradictory to our results. Wang *et al.* reported that AQP1 interacts with a destruction complex (including Wnt co-receptor LRP6, GSK3β, and Axin1) for β-catenin, thereby inhibiting the dissociation of this complex, enhancing β-catenin degradation, and suppressing Wnt signalling, whereas AQP1 deficiency enhances Wnt signalling^51^. *LRP4* has been shown to antagonize canonical Wnt signalling *in vitro* by competing with LRP5 or LRP6^7^,^17^,^52^,^53^. Therefore, the downregulation of AQP1 expression due to *Lrp4* deficiency may further enhance Wnt signalling.

We previously reported that *Lrp4* protein expression was higher in the forebrains of adult rats and in cultured rat cortical neurons^5^ than in other tissues. This finding was confirmed in the present study, although the expression of *LRP4* in the adult mouse brain was lower than in foetal organs (Fig. 1d). AQP1, which has been detected primarily in epithelial cells of the choroid plexus, is proposed to play a role in cerebrospinal fluid formation^54^, suggesting that *LRP4* may be involved in water homeostasis in the brain. *LRP4* was also found to be expressed in cultured astrocytes (Fig. 1e). *LRP4* may affect AQP9 involvement in the utilization of L-lactate for retinal neuronal survival^55^ and in astrocyte glucose metabolism by facilitating glycerol diffusion^56^. Recently, *Lrp4* was reported to play a role in hippocampal function^57^. The relationship between *LRP4* and AQP1/AQP9 in the central nervous system should be investigated in future studies.

In summary, foetal swallowing and breathing-like movements were found to be compromised in *Lrp4*^−/−^ foetuses. In addition, loss of *Lrp4* was observed to result in downregulation of the expression of AQP9 and AQP1, contributing to the regulation of amniotic fluid volume via excretion of amniotic fluid through the placenta and foetal membrane in the perinatal period. These results suggest that amniotic fluid removal was compromised in *Lrp4*^−/−^ foetuses, resulting in polyhydramnios despite the impaired urine production in these foetuses. Therefore, we conclude that amniotic fluid removal plays an essential role in regulating the volume of amniotic fluid.

## Methods

### Mouse Strains

Mouse *Lrp4* genomic clones were isolated from the RPCI-24 BACPAC library (C57BL/6J male; Children’s Hospital Oakland Research Institute) using mouse *Lrp4* cDNA as the probe. The 11,947-base genomic region, from 5,701 bases upstream of exon 1 to 6,125 bases downstream of exon 1 of the mouse *Lrp4* gene, was used to construct the targeting vector (Fig. 1a). The short- and long-arms of the targeting vector were 5,944 and 6,003 bases, respectively. A neomycin-resistance cassette (neo), the *PGK* promoter-driven neomycin-resistance gene^58^ with a *FRT* site at the 5’ end and a *FRT/loxP* site at the 3’ end, was inserted between the short- and long-arms. A second *loxP* site was inserted 1,527 bases upstream of exon 1 in the short-arm. The *diphtheria* toxin-A (DT-A) gene^59^ was introduced for negative selection. The targeting vector was linearized with *Xho*I and electroporated into the C57BL/6J mouse ES cell line BRUCE-4 and subsequently selected using G418. Positive clones were identified by PCR in order to confirm the existence of neo and *loxP* sites between the short-arm and exon 1, and verified homologous recombination by a Southern blot analysis. Positive clones were further analyzed by Cre- and Flp-recombinase treatments to confirm the correct removal of the sequence between two *loxP* sites and the sequence between two *FRT* sites, respectively. These two clones were used to create chimeric mice by a blastocyst injection, and chimeric mice were generated from one clone. The resultant male chimeras were mated with female CAG-cre transgenic mice, which express Cre recombinase in mature oocytes^60^. Some of the newborn mice were found to carry the deleted allele lacking *Lrp4* exon 1 as well as the neo (*Lrp4*^+/−^). Homozygous (*Lrp4*^−/−^) mice were obtained by intercrossing heterozygous (*Lrp4*^+/−^) mice overnight. Noon on the day on which the vaginal plug was found was counted as E0.5. PCR using genomic DNA extracted from tail clips was performed for genotyping. The null allele (PCR product size 375 bp) and wild-type allele (PCR product size 411 bp) were selectively amplified using the primers sets *Lrp4* nullF and *Lrp4* nullR, and *Lrp4* wtF and *Lrp4* wtR (Supplementary Table S2), respectively. Mice were maintained on a C57BL/6JJcl (CLEA Japan) background. This study was carried out in strict accordance with the recommendations in the Guidelines for Proper Conduct of Animal Experiments (Science Council of Japan). The protocol was approved by the Committee on the Ethics of Animal Experiments of Shinshu University (Permit Number: 200057, 230073 and 270015). All surgery was performed under sevoflurane inhalation anesthesia (Mylan Inc.), and all efforts were made to minimize suffering.

### Antibodies

Rabbit anti-LRP4 antisera were raised against the thioredoxin (Trx)-His-S-tagged fusion protein, Trx-His-S-mLRP4C161, which contained amino acids 1745–1905 of the C–terminal region of mouse *LRP4*. Antisera were pre-cleared on a HiTrap NHS-activated HP column (GE Healthcare) coupled with the Trx-His-S protein and purified on a HiTrap NHS-activated HP column coupled with Trx-His-S-mLRP4C50, which contained amino acids 1856–1905 of the C–terminal region of mouse *LRP4*, and the purified antibodies were termed *LRP4*C50. The goat anti-surfactant protein A (sc-7699) and rabbit anti-AQP1 (sc-20810) were purchased from Santa Cruz Biotechnology, while the mouse anti-glyceraldehyde-3-phosphate dehydrogenase (MAB374) was from Chemicon International, Inc.

### Western blotting

Foetal tissues (forebrain, heart, lungs, stomach, liver, intestine, kidneys, diaphragm, placenta, and foetal membrane) and maternal tissues (forebrain and myometrium) were homogenized in a 10-fold volume (ml)/weight (g) of RIPA buffer (50 mM Tris [pH7.5], 150 mM NaCl, 1% NP-40, 0.5% deoxycholic acid sodium salt, and 0.1% sodium dodecyl sulfate [SDS]) supplemented with the protease inhibitor CelLytic^TM^M (Sigma). Insoluble materials were removed by centrifugation (15,800 g for 30 min). Protein concentrations were determined using Pierce^®^ 660 nm Protein Assay Reagent (Thermo Fisher Scientific Inc.). Tissue lysates and amniotic fluid were analyzed by SDS-polyacrylamide gel electrophoresis (SDS-PAGE) and blotted onto PVDF membranes. Antibodies (LRP4C50, sc-7699 or sc-20810) were diluted in Can Get Signal^®^ (TOYOBO) and used in the Western blot analysis. Western blots were visualized and quantitatively analyzed using the Densitograph AE-6930 Lumino CCD and Lane analyzer 10H (ATTO Corp.). The mean of *Lrp4*^+/+^ and *Lrp4*^+/−^ fetuses born to the same mother was set as 1.

### Measurements of foetus, placenta, and amniotic fluid weights

On E13.5, 17.5, and 18.5, pregnant mice were euthanized by cervical dislocation. The abdomen was opened, the uterus was removed, and individual gestational sacs were carefully separated. The fluids including blood were wiped from the sac surface using a cotton swab, and each gestational sac was weighed before rupturing the amniotic membrane. After rupturing the amniotic membrane and the absorption of amniotic fluid, the foetus, placenta, and attachment were weighed. The amniotic fluid weight was calculated by the difference in the weight.

### Determinations of amniotic fluid osmolality and concentrations of electrolytes

On E18.5, amniotic fluid was aspirated from gestational sacs using a tuberculin syringe with a 20-gauge needle before opening the fetal membranes. Amniotic fluid was centrifuged for 10 min at 2,700 *g* and 20 min at 15,800 *g* to remove cellular debris. When the recovered fluid was more than 50 μl, the amniotic fluid was just used to determine osmolality and the concentrations of electrolytes, otherwise the amniotic fluid from the same genotype littermates was mixed and used in experiments. Fifty microliters of amniotic fluid was diluted to 200 μl with MilliQ water. Amniotic fluid osmolality was measured by freezing point depression (OSMO Station OM-6050, ARKRAY Inc.). Na^+^, K^+^, and Cl^−^ concentrations were determined with JCA-BM6050 (JEOL Ltd.).

### India ink injection into the amniotic cavity

On E17.5 and E18.5, pregnant mice were anesthetized and their abdomens were opened. In order to evaluate foetal breathing-like movements and swallowing, 100 μl of India ink (BOKUTEKI, Kuretake Co., Ltd.) was injected into the amniotic cavity of embryos using an insulin syringe with a pre-attached 29-gauge needle (ss-05M2913, TERUMO). After 30 min, the amniotic membrane was ruptured, and the foetus and placenta were washed in PBS^(−)^ and fixed in 4% paraformaldehyde-0.1M sodium phosphate buffer, pH7.4 for 1–5 days. BOKUTEKI is aqueous carbon black dispersion (average particle diameter, 150 nm) with polyvinyl alcohol as a protective colloid agent. Brilliant Blue FCF or Coomassie Brilliant Blue was initially used as the dye. However, these dyes had the following disadvantages over India ink: the foetal body was stained, these dyes were easily diffused from the lungs and stomach, and the contrast with lung tissue was lower.

### RNA isolation, and classic and quantitative RT-PCR

Total cellular RNA was extracted from the placenta and foetal membrane using ISOGEN (Nippongene) according to the manufacturer’s instructions. RNA concentrations were determined by spectrophotometry at an absorbance of 260 nm. cDNA in a 10-μl reaction was synthesized from 0.5 μg of RNA using ReverTraAce^®^ qPCR RT Master Mix with gDNA Remover (TOYOBO) according to the manufacturer’s instructions. PCR amplification was performed using established primers (Supplemental Table S3 online). All primers were first checked for their ability to specifically amplify defined mRNA regions. Classic RT-RCR for aquaporins was performed in a reaction mixture (5 μl) containing a 0.5-μl aliquot of cDNA, 0.5 μM of each primer, and KAPA Taq Extra HotStart (KAPA Biosystems) on an iCycler™ (Bio-Rad laboratories) with initial denaturation at 95 °C for 3 min, followed by 33 cycles of 95 °C for 15 s, 60 °C annealing for 15 s, and extension at 72 °C for 15 s. PCR products were run on a 6% acrylamide gel, stained with ethidium bromide and visualized using a myECL Imager (Thermo Fisher Scientific Inc.). Quantitative RT-PCR (qPCR) for the expression of *Lrp4*, aquaporins, SP-A, and β-actin (as the endogenous reference^35^) was conducted in a reaction mixture (10 μl) containing a 1-μl aliquot of cDNA, 0.4 μM of each primer, and SYBR^®^ *Premix Ex*Taq™II Tli RNaseH Plus (Takara Bio) on a StepOnePlus™Real Time PCR System (Thermo Fisher Scientific Inc.) with initial denaturation at 95 °C for 30 s, followed by 40 cycles of 95 °C for 5 s and 60 °C for 30 s. After each RT-PCR experiment, data were analyzed to select a threshold level of fluorescence in the linear phase of the PCR product accumulation. The difference in the threshold number of cycles between aquaporins or SP-A and β-actin (ΔC_t_) for each well was used for statistical testing.

### Statistical analysis

Quantified values were analyzed using the Prism 6.0 software program (GraphPad). Data on two groups were tested for normal distribution (F-test), and significance was analyzed using the Student’s *t*-test with or without Welch’s correction. Data on three groups were tested for normal distribution using the Brown-Forsythe test. When normality was confirmed, data were analyzed using a one-way analysis of variance. When the interaction was significant, multiple comparisons were made using Tukey-Kramer’s *post hoc* test. When normality was rejected, data were analyzed using the Kruskal-Wallis rank test, and multiple comparisons were made by Dunn’s *post hoc* test. Significance was accepted at *P* < 0.05. All values are expressed as means ± SD.

## Additional Information

**How to cite this article**: Tanahashi, H. *et al.* Polyhydramnios in *Lrp4* knockout mice with bilateral kidney agenesis: Defects in the pathways of amniotic fluid clearance. *Sci. Rep.* **6**, 20241; doi: 10.1038/srep20241 (2016).

## Supplementary Material

Supplementary Information

## Figures and Tables

**Table 1:** Changes in amniotic fluid (AF), foetal, and placental weights (mg) at E13.5, E17.5 and E18.5.

	wild-type	*Lrp4*^+/−^	*Lrp4*^−/−^
E13.5			
AF	150.1 ± 7.6 (n = 5)	162.4 ± 13.8 (n = 14)	159.5 ± 4.0 (n = 5)
Foetus	103.2 ± 10.6 (n = 5)	108.8 ± 5.7 (n = 14)	108.2 ± 4.5 (n = 5)
Placenta	70.4 ± 8.5 (n = 5)	67.8 ± 16.8 (n = 14)	68.8 ± 11.9 (n = 5)
E17.5			
AF	180.2 ± 52.0 (n = 9)	218.6 ± 36.7 (n = 18)	268.3 ± 44.1 (n = 11)*2^,^ @
Foetus	835.5 ± 194.4 (n = 9)	764.3 ± 159.1 (n = 18)	775.8 ± 128.9 (n = 11)
Placenta	111.8 ± 25.6 (n = 9)	103.9 ± 19.7 (n = 18)	107.7 ± 16.1 (n = 11)
E18.5			
AF	124.0 ± 36.7 (n = 30)	126.8 ± 37.0 (n = 49)	351.2 ± 50.1 (n = 24)*3^,^ @^3^
Foetus	1112.9 ± 104.1 (n = 30)	1109.7 ± 103.9 (n = 49)	1073.8 ± 62.0 (n = 24)
Placenta	106.0 ± 14.0 (n = 30)	108.5 ± 16.9 (n = 49)	111.9 ± 16.2 (n = 24)
E18.5 *Lrp4*^−/−^	Morphologically Normal kidney	Unilateral kidney Agenesis	Bilateral kidney Agenesis
AF	365.4 ± 48.6 (n = 7)*3^,^@^3^	339.7 ± 46.7 (n = 9)*3^,^@^3^	351.8 ± 58.0 (n = 8)*3^,^@^3^

Embryos at E13.5, E17.5, and E18.5 were obtained from a total of three, seven, and seventeen dam, respectively. Amniotic fluid weights of E18.5 *Lrp4*^−/−^ embryos were divided into three groups by kidney agenesis. Values are reported as the mean ± SD (mg). *^2^*P* < 0.001 versus the wild-type, *^3^*P* < 0.0001 versus the wild-type, @*P* < 0.05 versus *Lrp4*^+/−^, @^3^*P* < 0.0001 versus *Lrp4*^+/−^ by Tukey-Kramer’s *post hoc* test after a one-way analysis of variance. No significant differences were observed among the three groups of *Lrp4*^−/−^ embryos.

**Table 2:** Changes in amniotic fluid osmolality and amniotic fluid sodium, potassium, and chloride concentrations at E18.5.

	wild-type	*Lrp4*^+/−^	*Lrp4*^−/−^
Osmolality (mOsm/L)	348 ± 13 (n = 39)	348 ± 16 (n = 58)	335 ± 14 (n = 66)*2^,^ @^2^
Sodium (mM)	147 ± 6 (n = 39)	145 ± 10 (n = 58)	152 ± 8 (n = 66)*1^,^ @^3^
Potassium (mM)	12.6 ± 3.2 (n = 39)	13.7 ± 4.0 (n = 58)	8.9 ± 1.2 (n = 66)*3^,^ @^3^
Chloride (mM)	129 ± 5 (n = 39)	127 ± 10 (n = 58)	129 ± 6 (n = 66)
	***Lrp4*^−/−^**		
	**morphologically normal kidney**	**unilateral kidney agenesis**	**bilateral kidney agenesis**
Osmolality (mOsm/L)	336 ± 15 (n = 22)*, @	340 ± 17 (n = 28)	328 ± 12 (n = 16)*2^,^ @^2^
Sodium (mM)	151 ± 7 (n = 22)@	152 ± 9 (n = 28)*, @^2^	153 ± 7 (n = 16)*,@
Potassium(mM)	8.9 ± 1.2 (n = 22)*3^,^@^3^	8.6 ± 1.5 (n = 28)*3^,^@^3^	7.9 ± 1.6 (n = 16)*3^,^@^3^
Chloride (mM)	128 ± 5 (n = 22)	130 ± 7 (n = 28)	129 ± 7 (n = 16)

*Lrp4*^−/−^ foetuses were divided into three groups by kidney agenesis. Values are reported as the mean±SD. *P < 0.05 versus the wild-type, *^1^*P* < 0.01 versus the wild-type, *^2^*P* < 0.001 versus the wild-type, @*P* < 0.05 versus *Lrp4*^+/−^, @^2^*P* < 0.001 versus *Lrp4*^+/−^, @^3^*P* < 0.0001 versus *Lrp4*^+/−^ by Tukey-Kramer’s *post hoc* test after a one-way analysis of variance.

**Table 3:**
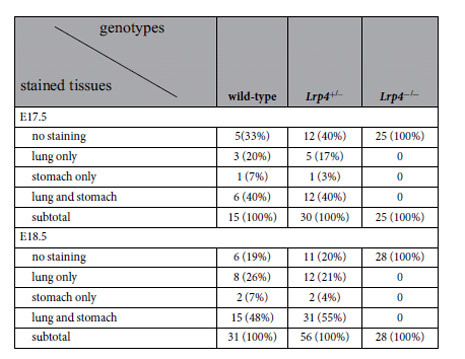
Indian ink accumulation in the lung and/or stomach after injection of the ink into the amniotic cavity of embryos at E17.5 and E18.5.

Foetuses at E17.5 and E18.5 were obtained from a total of 12 and 19 dam, respectively.

**Table 4:** Expression of AQP1 and AQP9 mRNA in the foetal membranes and placentas in wild-type and *Lrp4* ^−/−^ mice.

Groups (stage, tissues, AQPs)	Cases	∆Ct^a^	∆∆Ct^b^	FC^c^ (2^−∆∆Ct^)
E13.5, foetal membrane, AQP9	wild-type (n = 13)	14.76 ± 0.17	0 ± 0.17 1.00	(0.89–1.13)
	*Lrp4*^−/−^ (n = 7)	15.63 ± 0.35	0.87 ± 0.35	0.55 (0.43–0.70)*
E13.5, placenta, AQP1	wild-type (n = 13)	6.67 ± 0.31	0 ± 0.31	1.00 (0.80–1.24)
	*Lrp4*^−/−^ (n = 7)	6.79 ± 0.47	0.12 ± 0.47	0.92 (0.66–1.27)
E17.5, foetal membrane, AQP9	wild-type (n = 17)	13.17 ± 0.21	0 ± 0.21	1.00 (0.86–1.16)
	*Lrp4*^−/−^ (n = 25)	13.84 ± 0.18	0.67 ± 0.18	0.63 (0.55–0.71)*
E17.5, placenta, AQP1	wild-type (n = 17)	5.41 ± 0.24	0 ± 0.24	1.00 (0.85–1.18)
	*Lrp4*^−/−^ (n = 26)	6.14 ± 0.17	0.74 ± 0.17	0.60 (0.53–0.68)*
E18.5, foetal membrane, AQP1	wild-type (n = 24)	9.12 ± 0.12	0 ± 0.12	1.00 (0.91–1.09)
	*Lrp4*^−/−^ (n = 24)	9.16 ± 0.17	0.04 ± 0.13	0.97 (0.86–1.09)
E18.5, foetal membrane, AQP9	wild-type (n = 24)	14.25 ± 0.13	0 ± 0.13	1.00 (0.91–1.09)
	*Lrp4*^−/−^ (n = 24)	15.52 ± 0.15	1.27 ± 0.15	0.41 (0.22–0.75)*3
E18.5, placenta, AQP1	wild-type (n = 24)	5.54 ± 0.11	0 ± 0.11	1.00 (0.93–1.08)
	*Lrp4*^−/−^ (n = 24)	6.25 ± 0.24	0.71 ± 0.24	0.61 (0.52–0.72)*
E18.5, placenta, AQP9	wild-type (n = 24)	11.52 ± 0.10	0 ± 0.10	1.00 (0.93–1.07)
	*Lrp4*^−/−^ (n = 23)	11.52 ± 0.11	0 ± 0.11	1.00 (0.93–1.08)

Ct is the cycle number at a threshold level of fluorescence in the linear phase of PCR product accumulation. Values are reported as means ± SD.

^a^The ΔCt value was determined by subtracting the average β-actin Ct value from the average AQPs Ct value.

^b^ΔΔCt values were calculated by subtracting the mean ΔCt value in wild-type mice from the mean ΔCt value in *Lrp4*^*−/−*^ mice.

^c^The calculation of fold changes (FC) was performed based on the formula FC = 2^−ΔΔCt^ in order to calculate normalized gene expression FC in *Lrp4*^*−/−*^ mice samples relative to wild-type mice samples. The range given for AQP1 or AQP9 relative to wild-type was determined by evaluating the expression: (2^−∆∆Ct+SD^–2^−∆∆Ct-SD^). *P < 0.05 versus wild-type mice, *^3^*P* < 0.0001 versus wild-type mice by the Student’s *t*-test. An *Lrp4* deficiency reduced the expression levels of AQP1 protein in the placentas appreciably (Supplemental Fig. S5 online). The expression of AQP3, AQP7, and AQP8 in the foetal membranes and placentas in *Lrp4*^*−/−*^ was not significantly different from that in wild-type mice (Supplemental Table S4 online).
