# Supplementary material for: Polyhydramnios in Lrp4 knockout mice with bilateral kidney agenesis: Defects in the pathways of amniotic fluid clearance
Source: Sci Rep. 2016 Feb 5;6:20241. doi: 10.1038/srep20241 (PMC4742865; doi:10.1038/srep20241)
Supplement: Supplementary Information [file srep20241-s1.pdf]

Supplementary Information

Polyhydramnios in *Lrp4* knockout mice with bilateral kidney agenesis: Defects in the pathways of amniotic fluid clearance

Hiroshi Tanahashi[1,2,*], Qing-Bao Tian[1], Yoshinobu Hara[3], Hiroyuki Sakagami[3], Endo Shogo[4] and Tatsuo Suzuki[1,2]

[1]Department of Neuroplasticity, Institute of Pathogenesis and Disease Prevention, Graduate School of Medicine, [2]Department of Biological Sciences for Intractable Neurological Diseases, Institute for Biomedical Sciences, Shinshu University, 3-1-1 Asahi, Matsumoto 390-8621, Japan; [3]Department of Anatomy, Kitasato University School of Medicine, 1-15-1, Kitasato, Sagamihara, Kanagawa 252-0374, Japan; [4]Research Team for Aging Neuroscience, Tokyo Metropolitan Geriatric Hospital and Institute of Gerontology, Sakae-cho, Itabashi, Tokyo 173-0015, Japan

[*]Correspondence should be addressed to H.T. (E-mail: tanahasi@shinshu-u.ac.jp, Tel: +81-263-37-2727, Fax: +81-263-37-2725)

Supplementary Methods.

Lung weight and total DNA determinations. The lungs of E18.5 foetuses were blotted, weighed (wet lung weight), lyophilized for 24 h, and reweighed (dry lung weight). The dried lungs were acid-hydrolyzed[S1]. Deoxypurine nucleotides in the crude sample were reacted with 3,5-diaminobenzoic acid dihydrochloride (Tokyo Chemical Industry Co., Ltd.) and formed a fluorescent compound. Fluorescence was determined using Multi-Mode Microplate Reader Synergy™4 (BioTek) with a 410 nm, 10 nm bandwidth, excitation filter and a 508 nm, 20 nm bandwidth, emission filter.

Determination of creatinine and urea nitrogen concentrations in amniotic fluid. Creatinine concentrations were determined by the quantitative colorimetric assay LabAssay™ Creatinine (Wako Pure Chemical Industries, Ltd.), using an improved Jaffe method. Urea nitrogen concentrations were determined by the quantitative colorimetric assay Urea N B (Wako Pure Chemical Industries, Ltd.), using a Urease-Indophenol

method. Since ammonia and bilirubin in amniotic fluid had positive influences, they were measured by preparing a reaction without urease and the background was then subtracted from urea nitrogen readings. Four- and twenty-microliters of amniotic fluid were used in the reactions of Urea N B and LabAssay™ Creatinine, respectively. Urea nitrogen concentrations (mg/dL of nitrogen) were converted to mmol/L of urea. In order to reduce the number of animals used in these measurements and facilitate analyses, samples from wild-type and heterozygous fetuses were both used as controls, as they were phenotypically indistinguishable, and then compared with samples from homozygous littermate foetuses.

Alcian Blue/Alizarin red staining[S2]**.** Newborn mice were dissected by removing the skins and internal organs, fixed in ethanol for 1 day, and stained for 4 days in a staining solution consisting of 0.015% Alcian blue 8GX (Sigma), 20% acetic acid, and 80% ethanol. After rinsing with 95% ethanol, specimens were placed into 2% KOH for 1 day until they become clear, and stained for 1 day in a staining solution consisting of 0.0075% Alizarin red S (Sigma) and 1% KOH for 1 day until bones become dark purple. The samples were rinsed 5 times in 1% KOH and 20% glycerol for 1 day each time. The skeletons were further cleaned through a gradient series of glycerol/ethanol (1:1, 4:1, 1day each step), and then stored in glycerol.

Cell lines and a primary culture of astrocytes. The mouse muscle myoblast cell line, C2C12, human hepatocellular carcinoma cell line, HEPG2, and mouse macrophage cell lines, RAW264.7 and J774A.1 (purchased from ATCC) were cultured in Dulbecco's Modified Eagle's Medium (DMEM) supplemented with 10% fetal bovine serum (FBS). The mouse embryonal carcinoma cell line, P19 was cultured in DMEM supplemented with 15% FBS. C2C12 cells were differentiated to myotubes by switching to differentiation medium, DMEM supplemented with 2% horse serum, and 0.2 mM ascorbic acid[S3]. A primary culture of astrocytes was obtained from mouse cerebrum at E18.5[S4] and astrocytes were confirmed by immunocytochemistry using the astrocyte marker, GFAP.

Supplementary Table S1 | Changes in amniotic fluid urea and creatinine concentrations at E18.5.

	control (wild-type, *Lrp4*$^{+/-}$)	*Lrp4*$^{-/-}$
Urea (mM)	9.53±2.13 (n=43)	6.56±1.57 (n=34)*^{3}
Creatinine (mM)	0.18±0.06 (n=43)	0.086±0.026 (n=34)*^{3}

	Lrp4$^{-/-}$		
	morphologically normal kidney	unilateral kidney agenesis	bilateral kidney agenesis
Urea (mM)	7.61±1.06 (n=8)*	6.97±1.63 (n=11)*^{2}	5.57±1.31 (n=18)*^{3}
Creatinine (mM)	0.12±0.2 (n=8)*^{1}	0.092±0.019 (n=11)*^{3}	0.067±0.009 (n=18)*^{3}

Lrp4$^{-/-}$ foetuses were divided into three groups by kidney agenesis. Values are reported as the mean±SD (mM). *P<0.05 versus the control (wild-type, *Lrp4*$^{+/-}$), *^{1}P<0.01 versus the control, *^{2}P<0.001 versus the control, *^{3}P<0.0001 versus the control by Tukey-Kramer's *post hoc* test after a one-way analysis of variance.

Supplementary Table S2 | Body and lung weights of E18.5 foetuses (wild-type, *Lrp4*$^{+/-}$ and *Lrp4*$^{-/-}$), and DNA content in their lungs (mean ± SD)

	wild-type (n=17)	*Lrp4*$^{+/-}$ (n=25)	*Lrp4*$^{-/-}$ (n=16)
Body weight (mg)	1075.3±74.6	1069.2±84.9	1039.7±67.9
Wet lung weight (mg)	38.2±5.2	37.9±7.7	28.1±4.5*
Wet lung/body weight (μg/mg)	35.6±4.0	35.5±5.8	26.9±3.6*
Dry lung weight (mg)	5.1±0.5	5.1±0.7	5.0±0.7
Dry lung/body weight (μg/mg)	4.7±0.4	4.7±0.4	4.8±0.7
Lung DNA (mg)	0.244±0.025	0.237±0.033	0.233±0.026
Lung DNA/body weight (μg/mg)	0.227±0.023	0.221±0.021	0.224±0.024

Foetuses were obtained from a total of eight litters. *P<0.05 versus the wild-type and versus *Lrp4*$^{+/-}$ mice by Tukey-Kramer's *post hoc* test after a one-way analysis of variance.

Supplementary Table S3 | Oligonucleotide primers used for genotyping (Lrp4 nullF and Lrp4 nullR for the Lrp4 null allele, Lrp4 wtF and Lrp4 wtR for the Lrp4 wild-type allele), preparing a probe for southern blot analysis (Lrp4F and Lrp4R), and qPCR of Lrp4 (Lrp4F2 and Lrp4R2), aquaporins, β-actin (ActbF and ActbR), and SP-A (SP-A F and SP-A R).

Lrp4 nullF	5'-AAAGTGACTGACCTTGCCAC-3'
Lrp4 nullR	5'-AGCGTCTTGGGTCTTCTTCCAC-3'
Lrp4 wtF	5'-AGACTGGTCGCTGCAGGACTC-3'
Lrp4 wtR	5'-CTCTCCGCAATCCACCCAAG-3'
Lrp4F	5'-CCTAGTTGGATCAAAACCATGCC-3'
Lrp4R	5'-TACGTGGGTTGAGAGCTTAGG-3'
Lrp4F2	5'-GAGGGGGCAGGAGGAAGATG-3'
Lrp4R2	5'-CAGTCCTTCACCTGGAGCAG-3'
Aqp0F	5'-CTTGATCAATGTGGGAACTTCGGTCTGCCTCCT -3'
Aqp0R	5'-GCTCTAGACAATGTCTGAATTCCATTGAT-3'
Aqp1F	5'-TGCGTTCTGGCCACCACTGAC-3'
Aqp1R2	5'-GCAGCCAGTGTAGTCAATCGG-3'
Aqp2F	5'-GCCATCCTCCATGAGATTACC-3'
Aqp2R	5'-ACCCAGTGATCATCAAACTTG-3'
Aqp3F	5'-CTGGACGCTTTCACTGTGGGC-3'
Aqp3R2	5'-AGTGCCGGCCAGTCGTGAAG-3'
Aqp4F	5'-CTGGAGCCAGCATGAATCCAG-3'
Aqp4R	5'-TTCTTCTCTTCCCACGGTCA-3'
Aqp5F	5'-CTCTGCATCTTCTCCTCCACG-3'
Aqp5R	5'-TCCTCTCTATGATCTTCCCAG-3'
Aqp6F	5'-TCTGTTCTGCCCTGGCCTGTG-3'
Aqp6R	5'-ACCGCCTGGCCAGTTGATGTG-3'
Aqp7F	5'-GAGTCGCTAGGCATGAACTCC-3'
Aqp7R2	5'-CAGTTGTTTCCGGCTCTGAAC-3'
Aqp8F3	5'-GTAGGACTGCTCATTAGGCTCC-3'
Aqp8R	5'-CCTCGACTTTAGAATCAGGCGG-3'
Aqp9F	5'-CCTTCTGAGAAGGACCGAGCC-3'
Aqp9R2	5'-AGAGCCACATCCAAGGACAATC-3'

Aqp11F	5’-GTTTTTAAAAGCCTTAACCTT-3’
Aqp11R	5’-TCCCTCATTTCTTAACTT-3’
Aqp12F	5’-GCCCAGTACTCTGCTGAAACTGTT-3’
Aqp12R	5’-CCCTAGGGGTTCGGTATTTGCTTT-3’
ActbF	5’-TGTATGCCTCTGGTCGTACC-3’
ActbR	5’-CAGGTCCAGACGCAGGATG-3’
SP-A F	5’-AAAGGGGGCTTCCAGGGTTTC-3’
SP-A R	5’-TCCTTGCAAGCTGAGGACTCC-3’

Supplementary Table S4 | Expression of AQP3, AQP7, and AQP8 mRNA in the foetal membrane and placenta in wild-type and *Lrp4*$^{-/-}$ mice

Groups (stage, tissues, AQPs)	Cases	ΔCt [a]	ΔΔCt [b]	FC [c] ($2^{-\Delta\Delta Ct}$)
E18.5, foetal membrane, AQP3	wild-type (n=24)	7.61±0.09	0±0.09	1.00 (0.94-1.06)
	Lrp4$^{-/-}$ (n=24)	7.53±0.04	0.08±0.04	1.06 (0.92-0.97)
E18.5, foetal membrane, AQP7	wild-type (n=24)	13.50±0.08	0±0.08	1.00 (0.94-1.06)
	Lrp4$^{-/-}$ (n=24)	13.53±0.07	0.03±0.07	0.98 (0.93-1.03)
E18.5, foetal membrane, AQP8	wild-type (n=24)	4.09±0.10	0±0.10	1.00 (0.93-1.07)
	Lrp4$^{-/-}$ (n=24)	3.89±0.08	-0.20±0.08	1.15 (1.09-1.21)
E18.5, placenta, AQP3	wild-type (n=24)	5.36±0.06	0±0.06	1.00 (0.96-1.04)
	Lrp4$^{-/-}$ (n=24)	5.36±0.05	0.01±0.10	1.00 (0.96-1.03)
E18.5, placenta, AQP7	wild-type (n=24)	9.90±0.12	0±0.12	1.00 (0.92-1.09)
	Lrp4$^{-/-}$ (n=24)	9.65±0.46	-0.26±0.46	1.19 (0.87-1.64)
E18.5, placenta, AQP8	wild-type (n=24)	4.69±0.13	0±0.13	1.00 (0.93-1.07)
	Lrp4$^{-/-}$ (n=24)	4.71±0.08	0.02±0.08	0.98 (0.93-1.04)

Ct is the cycle number at a threshold level of fluorescence in the linear phase of the PCR product accumulation. Values are reported as means ± SD. [a]The ΔCt value was determined by subtracting the average β-actin Ct value from the average AQPs Ct value. [b]ΔΔCt values were calculated by subtracting the mean ΔCt value in wild-type mice from the mean ΔCt value in *Lrp4*$^{-/-}$ mice. [c]The calculation of fold changes (FC) was performed based on the formula FC = $2^{-\Delta\Delta Ct}$ in order to calculate normalized gene expression FC in *Lrp4*$^{-/-}$ mice samples relative to wild-type mice samples. The range given for AQP3, AQP7, or AQP8 relative to wild-type was determined by evaluating the expression: ($2^{-\Delta\Delta Ct+SD}$ - $2^{-\Delta\Delta Ct-SD}$). The expression of AQP3, AQP7 and AQP8 in the foetal membrane and placenta in *Lrp4*$^{-/-}$ mice was not significantly different from that in wild-type mice, as evaluated using the Student's *t*-test.

Supplementary Figure S1 | (a) *Lrp4*$^{-/-}$ neonates showed severe cyanosis and died shortly after birth. *Lrp4*$^{-/-}$ neonates exhibited polysyndactyly. (b) Alcian Blue/Alizarin red staining of fore limbs (c) The lungs of newborn wild-type and *Lrp4*$^{-/-}$ littermates were dissected and placed in PBS$^{(-)}$. Wild-type and mutant lungs are indicated. (d) Lung size was markedly smaller in *Lrp4*$^{-/-}$ at E18.5 than in the wild-type and *Lrp4*$^{+/-}$, but was morphologically normal. (e) Microscopic comparisons of lungs from newborn wild-type and *Lrp4*$^{-/-}$ littermates. Wild-type alveolar spaces were expanded, indicating respiration; however, *Lrp4*$^{-/-}$ alveoli were still collapsed, and the septae were hypercellular. Sections were stained with hematoxylin/eosin.

¥^-@

(a)

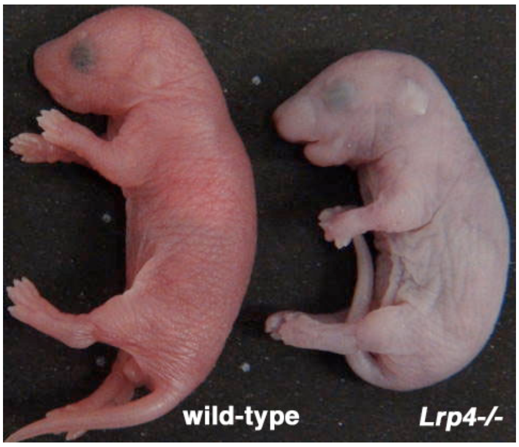

(b)

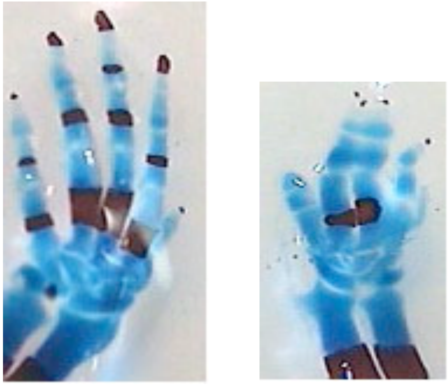

wild-type **_Lrp4-/-_**

(c)

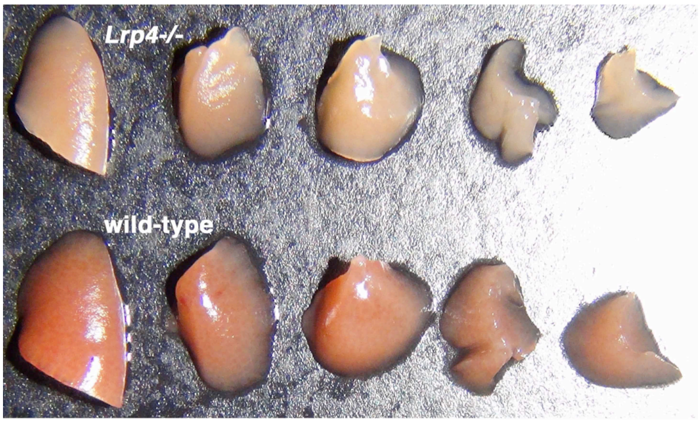

(d)

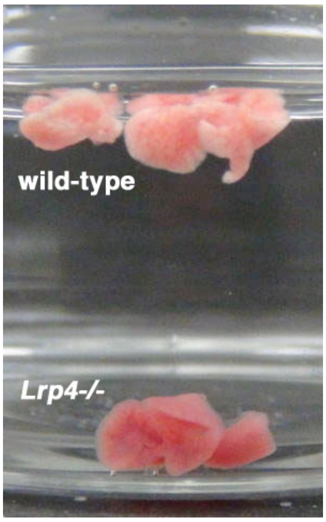

(e)

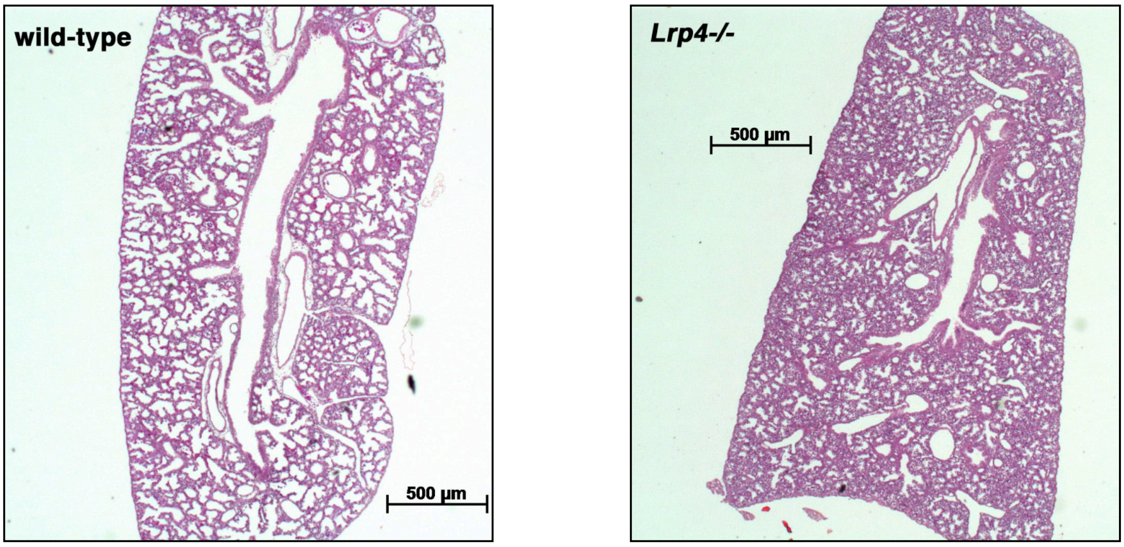

Supplementary Figure S2 | Successful targeting of the *Lrp4* gene was confirmed by a sequencing analysis. Sequences with yellow backgrounds and red backgrounds indicate a mouse genomic sequence and bacteriophage P1 *lox*P sequence, respectively. An underline and double underline indicate a Lrp4 null F primer sequence and anti-sense sequence of a Lrp4 null R primer, respectively. G : 1,594 bases upstream from the A site of the translation initiation ATG codon, T : 174 bases downstream from the A site of the translation initiation ATG codon (123 bases downstream from the end of exon 1)

```
5'-AAAGTGACTGACCTTGCCACACAAGCCAGAGAAATCCACTCTTTCCTAAGCTCTCA
ACCCACGTAAACCTCATCTGATTCTACTGCCGCCTTCAGGTTCCAGGAAAAATTACACA
GCCAGAAAATAGCTAGGCATTAGTTACCATGCATAACTTCGTATAATGTATGCTATAC
GAAGTTATTAGGTCTGAAGAGGAGTTTACGTCCAGTCTAGAGCGGCCCAATTGGTCGA
GCTCACTTCGAGCACCAACCCTGGCAGAGCGAGCGGAGGAAAGCAATAGCAGCCCCCGC
AGCTCTTCTAGCACGAGAGGGGACAGCGGTGGTGGGACTCGTCCCCGCTGTAGTCTGGG
CCGGGTGGAAGAAGACCCAAGACGCT-3'
```

Supplementary Figure S3 | Dissection of the E18.5 gastrointestinal tract of wild-type and *Lrp4*$^{-/-}$ foetuses. Judging by appearance, *Lrp4*$^{-/-}$ foetuses did not appear to have gastrointestinal atresia or stenosis. an: anus, ca: caecum, es: esophagus, st: stomach, to: tongue

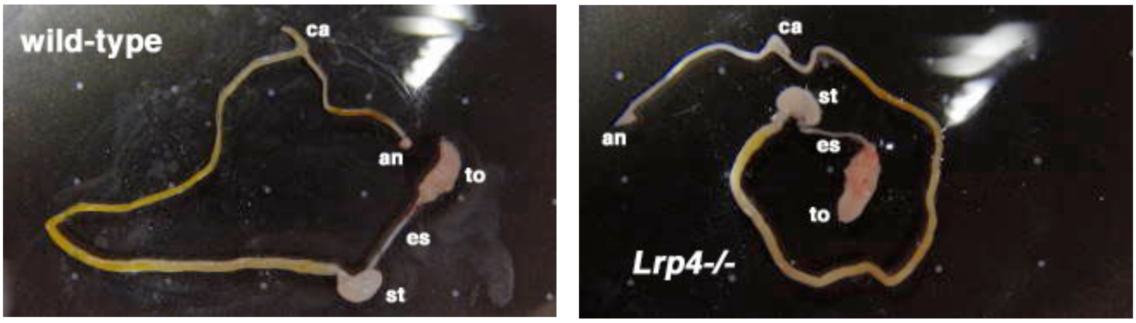

Supplementary Figure S4 | Expression of aquaporins in the placenta and foetal membrane at E18.5. (a) RT-PCR analysis of AQP transcripts in the placenta. (b) RT-PCR analysis of AQP transcripts in foetal membranes. bACT: β-actin

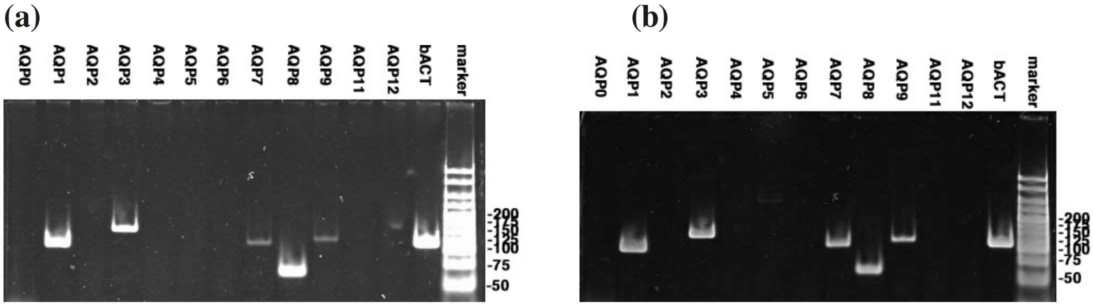

Supplementary Figure S5 | An *Lrp4* deficiency affected the expression of the AQP1 protein in the placenta. Placenta lysates (each 50 μg of protein) from wild-type (lanes 2, 3, 7, 8), and *Lrp4*$^{-/-}$ (lanes 1, 4, 5, 6) foetuses at E18.5 were analyzed by western blot analysis with anti-Lrp4 and anti-AQP1 antibodies. A promonant 28 kDa band reveals non-glycosylated AQP1 protein. Broad bands (30-50 kDa) may contain glycosylated AQP1 protein. After stripping, the same blots were analyzed using anti-GAPDH antibodies. Lanes 1-4, and lanes 5-8 were their respective littermates. An *Lrp4* deficiency reduced the expression levels of non-glycosylated AQP1 protein at E18.5 in

the foetal placentas appreciably. Cropped blots are shown (full-length blots are presented in Supplementary Fig. S6 online). w: wild-type, -/-: *Lrp4*$^{-/-}$

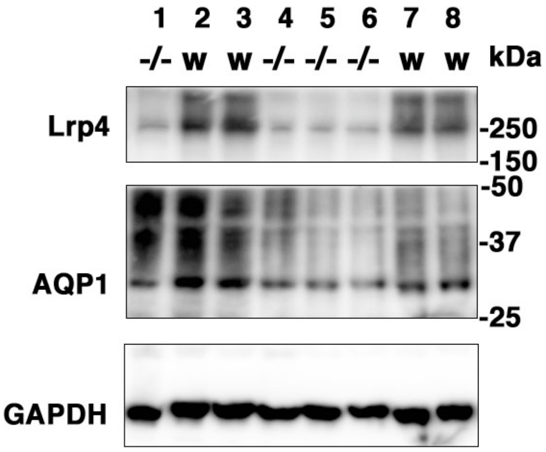

Supplementary Figure S6 | All non-cropped western blots are shown for the representative blots in the figures. Blots shown for Figure 1 (c-e), Figure 4 (a,c) and Figure S5.

Fig. 1c blot | stain: anti-Lrp4 | stain: anti-GAPDH

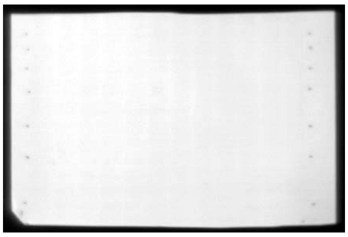
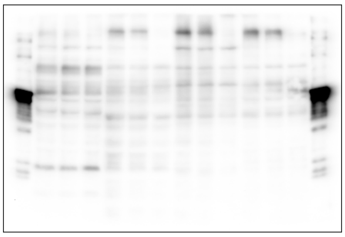
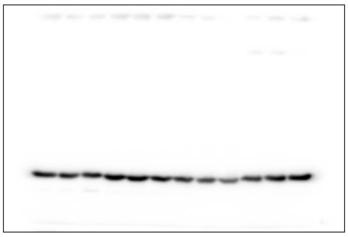

Fig. 1d blot | stain: anti-Lrp4 | stain: anti-GAPDH

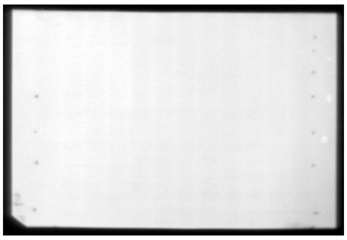
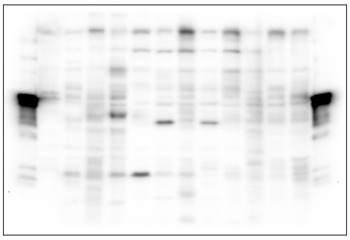
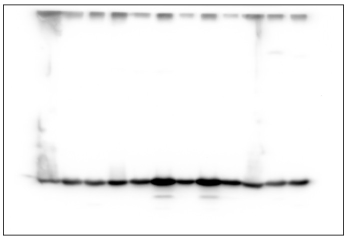

Fig. 1e blot stain: anti-Lrp4

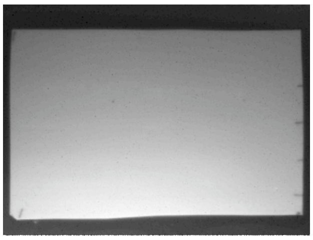

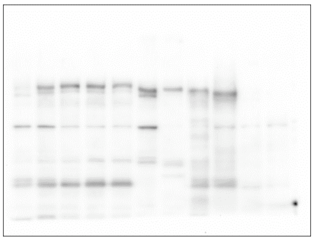

stain: anti-GAPDH

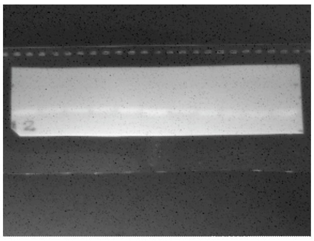

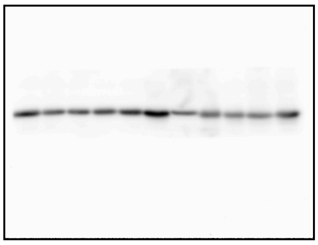

Fig. 4a blot stain: anti-SP-A

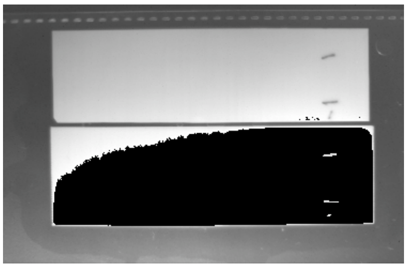

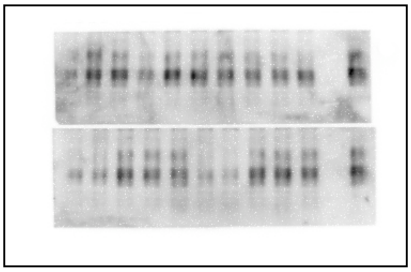

Fig. 4c blot stain: anti-SP-A

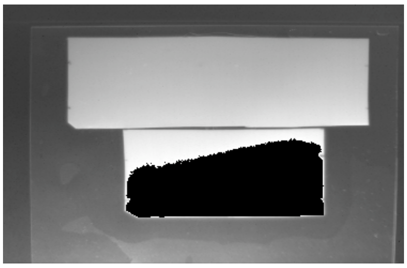

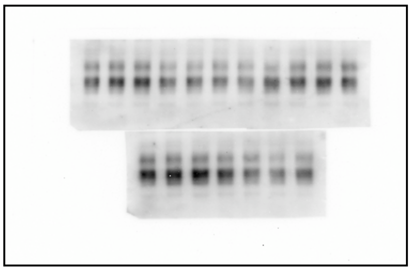

After stripping, the same blots were analyzed using anti-GAPDH.

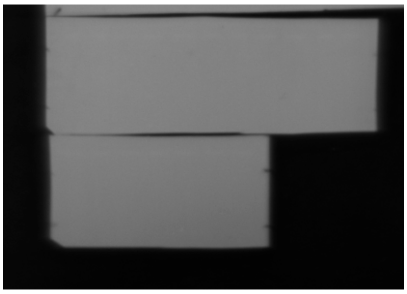

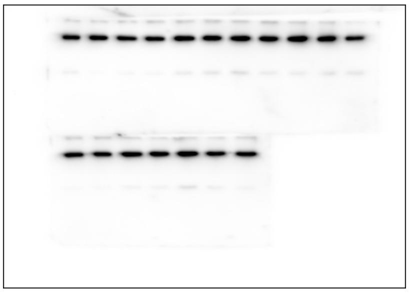

Fig. S5 blot

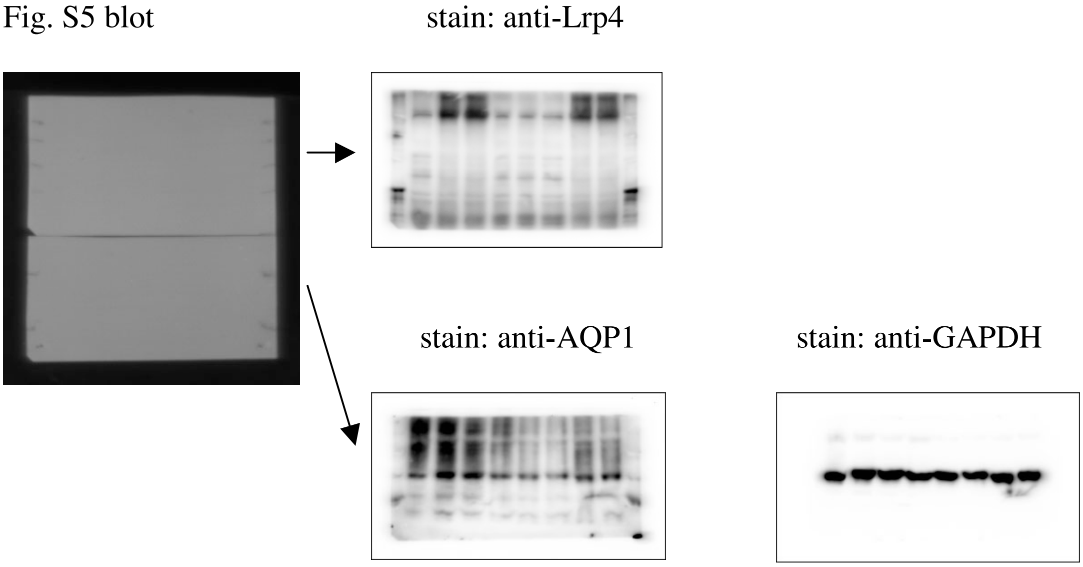

After stripping, the same blots were analyzed using anti-GAPDH.

Supplementary References

S1. Kissane, J. M. & Robins, E. The fluorometric measurement of deoxyribonucleic acid in animal tissues with special reference to the central nervous system. *J Biol Chem* **233**, 184-188 (1958).

S2. Inouye, M. Differential staining of cartilage and bone in mouse skeleton by alcian blue and alizarin Red S. *Congenital Anomalies* **16**, 171-173 (1976).

S3. Yaffe, D. & Saxel, O. Serial passaging and differentiation of myogenic cells isolated from dystrophic mouse muscle. *Nature* **270**, 725-727 (1977).

S4. de Vellis, J., Ghiani, C. A., Wanner, I. B. & Cole, R. Preparation of normal and reactive astrocyte cultures. In: Doering, L. C. ed. *Protocols for Neural Cell Culture fourth edition*. Humana Press, New York, 193-215 (2010).
